# Special Issue “Pediatric Viral Infection Long-Term Consequences”

**DOI:** 10.3390/v14020343

**Published:** 2022-02-08

**Authors:** Regina K. Rowe, Emma L. Mohr

**Affiliations:** 1 Department of Pediatrics, University of Rochester Medical Center, Rochester, NY 14642, USA; 2 Department of Pediatrics, University of Wisconsin-Madison, Madison, WI 53792, USA

This Special Issue was focused on advancing our understanding of the long-term consequences of pediatric viral infections. Viral infections in childhood can result in negative sequelae for months or years following resolution of acute infection. Many virus infections of childhood can result in long term sequelae including developmental deficits, chronic airway diseases, and immunomodulation. Despite the potential life-long impact of early virus infections in children, we have a poor understanding of the pathogenesis underlying post-viral sequelae, and an even poorer understanding of effective therapies. This Special Issue contains six original studies that contribute to the overall knowledge of the long-term consequences of pediatric viral infections.

Studies in this issue contributed to our knowledge of congenital Zika virus infection, with manuscripts utilizing animal models in rats and nonhuman primates, and a human clinical study. A new rat model of prenatal Zika virus infection described novel automated atlas-based segmentation of MR images and found that prenatal Zika virus infection alters the growth of brain regions throughout neonatal and juvenile ages [1]. A nonhuman primate model of prenatal Zika virus infection explored the impact of maternal Dengue virus immunity on neonatal development, identifying that maternal Dengue virus immunity exacerbates Zika-induced visual orientation and tracking deficits [2]. Finally, a human study investigated the impact of prenatal Zika virus exposure in children without microcephaly, and discovered that a small proportion of children without microcephaly still had higher frequencies of cognitive delay than controls [3].

In addition to the long-term sequelae of congenital Zika virus infection, we also learned about other common viruses which can have serious consequences in children. The acquisition of cytomegalovirus (CMV) by very-low-birthweight infants is incompletely characterized, and Hernandez-Alvarado, N et al. [4] found that higher CMV DNA levels in the breast milk were associated with an increased likelihood of CMV DNA in the infant’s blood. A human study investigated the rate of human papillomavirus transmission from mother to infant given the risk for recurrent respiratory papillomatosis in children with HPV infection, and found that 10–53% of newborns had HPV DNA detected in the nasopharynx [5]. Lastly, severe influenza infection can have long term health consequences in high risk children. Vaccination is the most effective intervention for prevention, but it is unclear if children with obesity mount as robust and protective responses as compared to normal weight children. Kainth et al. found that influenza vaccination provides similar cytokine and chemokine responses in normal weight and obese children, suggesting that a child’s weight does not alter the efficacy of vaccination [6].

We appreciate all the authors for their contributions to this Special Issue. Their dedication to elucidating the underlying pathophysiology for long-term consequences of pediatric viral infections is apparent. Together, we will continue to characterize, diagnose and improve the outcomes of the long-term consequences of pediatric viral infections. 
